# Endolysin NC5 improves early cloxacillin treatment in a mouse model of *Streptococcus uberis* mastitis

**DOI:** 10.1007/s00253-023-12820-w

**Published:** 2024-01-10

**Authors:** Niels Vander Elst, Julie Bellemans, Rob Lavigne, Yves Briers, Evelyne Meyer

**Affiliations:** 1 Laboratory of Gene Technology, Department of Biosystems, Faculty of Bioscience Engineering, KU Leuven, Kasteelpark Arenberg 21, 3001 Heverlee, Belgium; 2 Laboratory of Applied Biotechnology, Department of Biotechnology, Faculty of Bioscience Engineering, Ghent University, Valentin Vaerwyckweg 1, 9000 Ghent, Belgium; 3 Laboratory of Biochemistry, Department of Veterinary and Biosciences, Faculty of Veterinary Medicine, Ghent University, Salisburylaan 133, 9820 Merelbeke, Belgium

**Keywords:** Endolysin, Penicillin, Cloxacillin, *Streptococcus uberis*, Mouse model, Bovine mastitis

## Abstract

**Abstract:**

*Streptococcus uberis* frequently causes bovine mastitis, an infectious udder disease with significant economic implications for dairy cows. Conventional antibiotics, such as cloxacillin, sometimes have limited success in eliminating *S. uberis* as a stand-alone therapy. To address this challenge, the study objective was to investigate the VersaTile engineered endolysin NC5 as a supplemental therapy to cloxacillin in a mouse model of bovine *S. uberis* mastitis. NC5 was previously selected based on its intracellular killing and biofilm eradicating activity. To deliver preclinical proof-of-concept of this supplemental strategy, lactating mice were intramammarily infected with a bovine *S. uberis* field isolate and subsequently treated with cloxacillin (30.0 μg) combined with either a low (23.5 μg) or high (235.0 μg) dose of NC5. An antibiotic monotherapy group, as well as placebo treatment, was included as controls. Two types of responders were identified: fast (*n* = 17), showing response after 4-h treatment, and slow (*n* = 10), exhibiting no clear response at 4 h post-treatment across all groups. The high-dose combination therapy in comparison with placebo treatment impacted the hallmarks of mastitis in the fast responders by reducing (i) the bacterial load 13,000-fold (4.11 ± 0.78 Δlog_10_; *p* < 0.001), (ii) neutrophil infiltration 5.7-fold (*p* > 0.05), and (iii) the key pro-inflammatory chemokine IL-8 13-fold (*p* < 0.01). These mastitis hallmarks typically followed a dose response dependent on the amount of endolysin added. The current *in vivo* study complements our *in vitro* data and provides preclinical proof-of-concept of NC5 as an adjunct to intramammary cloxacillin treatment.

**Key points:**

*• Engineered endolysin NC5 was preclinically evaluated as add-on to cloxacillin treatment.*

*• Two types of mice (slow and fast responding) were observed.*

*• The add-on treatment decreased bacterial load, neutrophil influx, and pro-inflammatory mediators.*

**Supplementary Information:**

The online version contains supplementary material available at 10.1007/s00253-023-12820-w.

## Introduction

Bovine mastitis is defined as an inflammation of the udder caused by the propagation of pathogens in the mammary gland, of which *Streptococcus uberis* (*S. uberis*) is one of the foremost causative Gram-positive bacteria (de Jong et al. 2018). This major pathogen is hard to eradicate from dairy farms, given its dissemination in the dairy stable such as in straw (Klaas and Zadoks 2018). Moreover, *S. uberis* can evade early detection by the innate immune system of the mammary gland and is able to survive antibiotic treatment by persisting in a dormant state either inside bovine mammary epithelial cells (boMECs) or in a biofilm (Günther et al. 2016; Rainard et al. 2022). These virulence factors also pose a challenge for the current treatment that is based on the intramammary administration of antibiotics (Tamilselvam et al. 2006; Schönborn et al. 2017; Moliva et al. 2022). In addition, the use of antibiotics in livestock is increasingly questioned, with legislative authorities imposing restrictions (e.g., European Green Deal, WHO; EU Regulation on Veterinary Medicines 2019/06). To address this problem, our group has previously characterized the bacteriophage-derived endolysins PlySs2 and PlySs9 with potent bacteriolytic activity against *S. uberis* to complement the current intramammary antibiotic arsenal (Vander Elst et al. 2020b). Starting from these PlySs2 and PlySs9 wild types, we created a superior so-called third-generation variant named NC5 by means of the VersaTile technology that (i) kills *S. uberis* intracellularly in boMECs, (ii) eradicates *S. uberis* biofilm, and (iii) potentiates cloxacillin treatment in raw milk from *S. uberis*–infected dairy cows (Vander Elst et al. 2023b). Cloxacillin is a narrow-spectrum isoxazolyl penicillin with intrinsic resistance to β-lactamases that is frequently used intramammarily to prevent and treat Gram-positive bovine mastitis (Thomas et al. 2015; de Jong et al. 2018).

In Gram-positive mastitis, the pathogen-associated molecular patterns (PAMPs) are recognized by toll-like receptors (TLRs) on the mammary epithelium (Ezzat Alnakip et al., 2014; Rainard et al., 2022). This causes pro-inflammatory paracrine signaling through the epithelial production of cytokines, which triggers resident mammary ductal macrophages to create a positive feedback loop by upregulating tumor necrosis factor (TNF)-α, interleukin (IL)-1α, IL-1β, IL-6, and IL-8 as well as chitinase-3-like protein 1 (CHI3L1) (Breyne et al. 2018). Subsequently, IL-8 attracts circulating neutrophils into the mammary gland where they are activated and kept viable by granulocyte colony-stimulating factor (G-CSF) to inhibit bacterial pathogen growth by phagocytosis and the excretion of the iron chelator lipocalin-2 (LCN2) (Martin et al. 2021; Vander Elst et al. 2023a). Following this initial neutrophil invasion, macrophages are similarly attracted to the infection site in a later stage by monocyte chemoattractant protein-1 (MCP-1) and subsequently activated by macrophage colony-stimulating factor (M-CSF). It has been shown specifically for *S. uberis* that these ductal macrophages play a key role in pathogen recognition due to the inability of the mammary epithelium to detect its PAMPs (Günther et al. 2016). Still, the initial phase of the innate immune response upon infection with *S. uberis* compared to other mastitis pathogens was recently reported to be conserved in experimental mastitis (Salamon et al. 2023).

The present study aimed to evaluate if our previously reported promising *in vitro* activity of the top candidate engineered endolysin NC5 as add-on to cloxacillin can be extended *in vivo*. Therefore, the hallmarks of mastitis were monitored in the mammary glands of *S. uberis*–infected mice treated with this combination strategy, compared to cloxacillin as a monotherapy and placebo treatment. The hallmarks of mastitis are defined as the presence of (i) clinical symptoms (e.g., fever, redness, and swelling), (ii) an influx of neutrophils, (iii) pathogenic bacteria, and (iv) the increase of pro-inflammatory cytokines, chemokines, growth factors, and metabolites (Vander Elst et al. 2020a; Rainard et al. 2022).

## Materials and methods

### Bacterial strain, culture conditions, and infective inoculum

The selected bovine *S. uberis* mastitis isolate used in this study is available from the Belgian Co-ordinated Collections of Micro-organisms (BCCM) with accession number LMG 33250. The genomic content of this *S. uberis* isolate is available from the PubMLST database (https://pubmlst.org/; id number: 2796). Inoculum preparation was done by growing *S. uberi*s to stationary phase in brain heart infusion (BHI), after which cells were washed with Dulbecco’s phosphate buffered saline (dPBS) and diluted in dPBS to the desired concentration based on OD_600_ measurements (Beal et al. 2020). The inoculum consisted of approximately 10^3^ colony forming units (CFU) in 100 μL dPBS.

### Endolysin expression, purification, and therapeutic inoculum

Overnight, 5.0 mL cultures of transformed lipopolysaccharide (LPS)-deficient ClearColi™ BL21 (DE3) (Lucigen LGC, Germany) were poured into 2.0-L Erlenmeyer flasks filled with 0.5 L lysogeny broth (LB) and the addition of 50.0 μg/mL kanamycin as selection marker for the endolysin-harboring expression vector pVTD3 (Gerstmans et al. 2020). When the *Escherichia coli* obtained an optical density measured at 600 nm (OD_600_) of 0.6–0.8, protein expression was induced by addition of 1 mM isopropyl β-d-thiogalactoside (Carl Roth, Germany). After expression for 16 h at 16 °C, *E. coli* were pelleted, supernatant was decanted, and the pellet was stored at −80 °C until further processing. Resuspension in PBS containing 10 mM imidazole (pH 7.4) (Carl Roth, Germany), 1 mM phenylmethylsulfonyl fluoride (PMSF) (Carl Roth, Germany), and 1 mM DNase I (NEB, USA) preceded sonication on ice and centrifugation (20,000 *g*; 4 °C; 20 minutes (min)). The filter-sterilized lysate was subsequently applied to an ÄKTA pure™ protein purification system (Cytiva, USA). The tubing of this system was first lipopolysaccharide (LPS)-decontaminated by running 1 M NaOH (Chem-Lab, Belgium) at 1.0 mL/min during 2 h, followed by washing with ultrapure water (Merck Millipore, USA). The system and the attached nickel sepharose high performance column (Cytiva, USA) were then equilibrated by sterile Dulbecco’s phosphate buffered saline (dPBS) (Gibco, Thermo Fisher Scientific, USA) containing 10 mM imidazole (pH 7.4). Subsequently, the lysate was pumped over for this column to perform Ni-NTA chromatography. Washing of the column was done by dPBS and the addition of (i) 10 mM imidazole; (ii) 20 mM imidazole, 0.5 M NaCl (Carl Roth, Germany), and 0.1% Empigen BB (Merck, USA); and (iii) 50 mM imidazole, 0.5 M NaCl, and 10% glycerol (VWR, Belgium), all at pH 7.4. Finally, the target protein was eluted from the column with 500 mM imidazole, 0.5 M NaCl, and 10% glycerol in dPBS (pH 7.4). The eluted protein was fractioned as 1.0 mL aliquots in a sterile 96-deepwell plate (Thermo Fisher Scientific, USA). Fractions that contained the envisaged protein, as was evaluated by SDS-PAGE, were pooled, and buffer exchange to dPBS was performed using the Pierce™ Protein Concentrators PES with a molecular weight cutoff of 10 kDa (Thermo Fisher Scientific, USA). Finally, the protein was filter sterilized (PVDF membrane, 0.45 μm) by means of a syringe, and the protein concentration was determined by the Bradford protein assay (Bio-Rad, USA). The bacteriolytic activity of the protein was checked by performing a turbidity reduction assay against the *S. uberis* isolate used to infect the mice, as described (Vander Elst et al. 2020b). Endotoxins present in the protein sample were determined by the ToxinSensor Chromogenic LAL Endotoxin Assay Kit according to the manufacturer’s protocol (GenScript, The Netherlands). The protein was stored on ice at 4 °C until the day of the *in vivo* experiment.

### Ethics statement and animal procedures

All experimental procedures on mice were executed at the Faculty of Veterinary Medicine of Ghent University (Merelbeke, Belgium) and with approval of the ethics committee (approval number: 2021/096). Breeding pairs of female and male CD-1 mice (Envigo, The Netherlands) were allowed to mate for 2 weeks, after which the dams gave birth approximately 7 days later. Twelve days postpartum, the lactating dams were intraductally inoculated with a blunted 32-gauge pediatric needle in the fourth inguinal mammary gland pair with the infective or therapeutic inoculum (i.e., bilaterally in the left and right gland) after properly disinfecting the teat (Hibitane Plus, Belgium). These intraductal inoculations were always performed under general gas anesthesia, using a mixture of medical oxygen and isoflurane at 2.5–3.0% for induction and 1.5–2.0% for maintenance. The long-acting analgesic buprenorphine (Bupaq, Richter Pharma AG, Austria) was administered at 10 μg/kg intraperitoneally for post-surgical pain relief.

### Murine mammary gland collection and analyses

Mice received a cocktail of 100 mg/kg ketamine (Ketamidor, Ecuphar, Belgium) supplemented with 10 mg/kg xylazine (Xylazini Hydrochloridum, Val d’Hony-Verdifarm, Belgium) and were subsequently euthanized by cervical dislocation to harvest the mammary glands. These were retained for either (i) histology and immunohistochemistry or (ii) determination of the bacterial load and local inflammatory protein profiling.

(i) Harvested mammary glands were fixed for 24 h in 3.5% buffered formaldehyde for subsequent embedding in paraffin. Histology required deparaffinization and rehydration of 5-μm sections followed by short (5 min) incubation in hematoxylin and eosin (H&E) staining buffers. Neutrophils and macrophages were stained by immunohistochemistry (IHC) for the neutrophil marker Ly6G (anti-Ly6G, BioLegend, USA) and macrophage marker Iba-1 (anti-Iba-1, Abcam, The Netherlands) on paraffin sections. Deparaffinized and hydrated tissue slides were incubated in citrate buffer (i.e., 10 mM tri-sodium citrate, 0.05% Tween 20, pH 6.0) in a decloacking chamber under pressure at 95 °C for 30 min. Following endogenous peroxidase blocking (3% H_2_O_2_ in methanol for 10 min at 22 °C), sections were incubated with primary rat anti-mouse Ly6G antibody (diluted 1:1000 in antibody diluent (Biocare Medical, USA)) or primary rabbit anti-mouse Iba-1 (diluted 1:2000) for 1 h at 22 °C. Rat-on-mouse or rabbit-on-rodent HRP polymer (Biocare Medical, USA) served as secondary antibody on the tissue sections for 30 min at 22 °C. Detection of the staining was performed by applying 3,3′-diaminobenzidine (Biocare Medical, USA) on the tissue sections for 10 min. For microscopic evaluation, tissue sections were rehydrated and mounted with a cover glass. Throughout the staining procedure, slides were kept in a humidified box and washed between each incubation step with Tris-buffered saline (Biocare Medical, USA). IHC for the endolysin was identically performed by antigen retrieval in Tris-EDTA buffer (10 mM Tris, 1 mM EDTA, 0.05% Tween 20, pH 9.0) and applying rabbit anti-hexa-histidine (diluted 1:1600) (Abcam, The Netherlands) as primary detection antibody. ImageJ was used to quantify positive staining (i.e., color deconvolution and automatic counting of % area), as described previously (Almeida et al. 2012; Vander Elst et al. 2020a; Steenbrugge et al. 2022).

(ii) To quantify the bacterial loads, the isolated mammary glands were homogenized using a TissueRuptor (Qiagen, Germany) and 1:10 serially diluted in dPBS followed by plating on BHI with the addition of 15.0 g/L agar (Oxoid, Belgium). Mammary glands were initially weighed, and bacterial load was expressed as log_10_(CFU/g tissue). Harvested mammary glands were always kept cold on melting ice. The local host response to the experimental mammary gland infection and subsequent therapy was investigated by inflammatory protein profiling of mammary gland lysates, obtained by mixing the homogenates with 300 μL caspase lysis buffer (CLB) containing protease inhibitors, and subsequent centrifugation (12,000 g; 4 °C; 20 min). The protein concentration of these lysates was determined using the Bradford protein assay. Standardization was done by dilution in CLB to equal protein concentrations. Levels of the selected inflammatory mediators TNF-α, MCP-1, M-CSF, and G-CSF as well as IL-1α, IL-1β, IL-6, IL-8, and IL-10 were determined on mammary gland lysates using a ProcartaPlex Immunoassay (Thermo Fisher Scientific, USA). Quantification of CHI3L1 and LCN2 was performed by ELISA (Bio-Techne, Minneapolis, MN, USA).

### Sample size calculation and statistical analysis

Sample size was calculated to aim a post-experimental analysis of the variance (ANOVA), respecting a type I error of 5%, power of 80%, mean difference of 1.5 log_10_, and variance of 0.60 or 0.50 (latter two for comparison between treatment groups or for comparison with the placebo group, respectively). These values were based on the previous observations made in the *S. uberis* mastitis mouse model (Demon et al. 2013). The sample size calculation suggested to use ≥ 6 mice for comparison between treatment groups and ≥ 4 mice for comparison with the placebo group. Data were analyzed using GraphPad Prism (version 9.5.1) to calculate *p*-values and determine statistically significant differences (*p* < 0.05). The average per animal was used to avoid a blocking effect originating from mice that were either once or twice represented, given that sometimes either the left or right mammary gland was retained for microscopy. Two groups were compared with an unpaired, two-tailed *t*-test, whereas multiple groups were compared by ANOVA and a Holm-Šídák post hoc test. Data were confirmed to be normally distributed by quantile-quantile plotting the residuals (*n* ≤ 6) or by performing an Anderson-Darling test (*n* > 6). Outliers were removed by means of the ROUT method with *Q* set to 5%, and this occasional removal is mentioned in the figure legends.

## Results

**Fig. 1:**
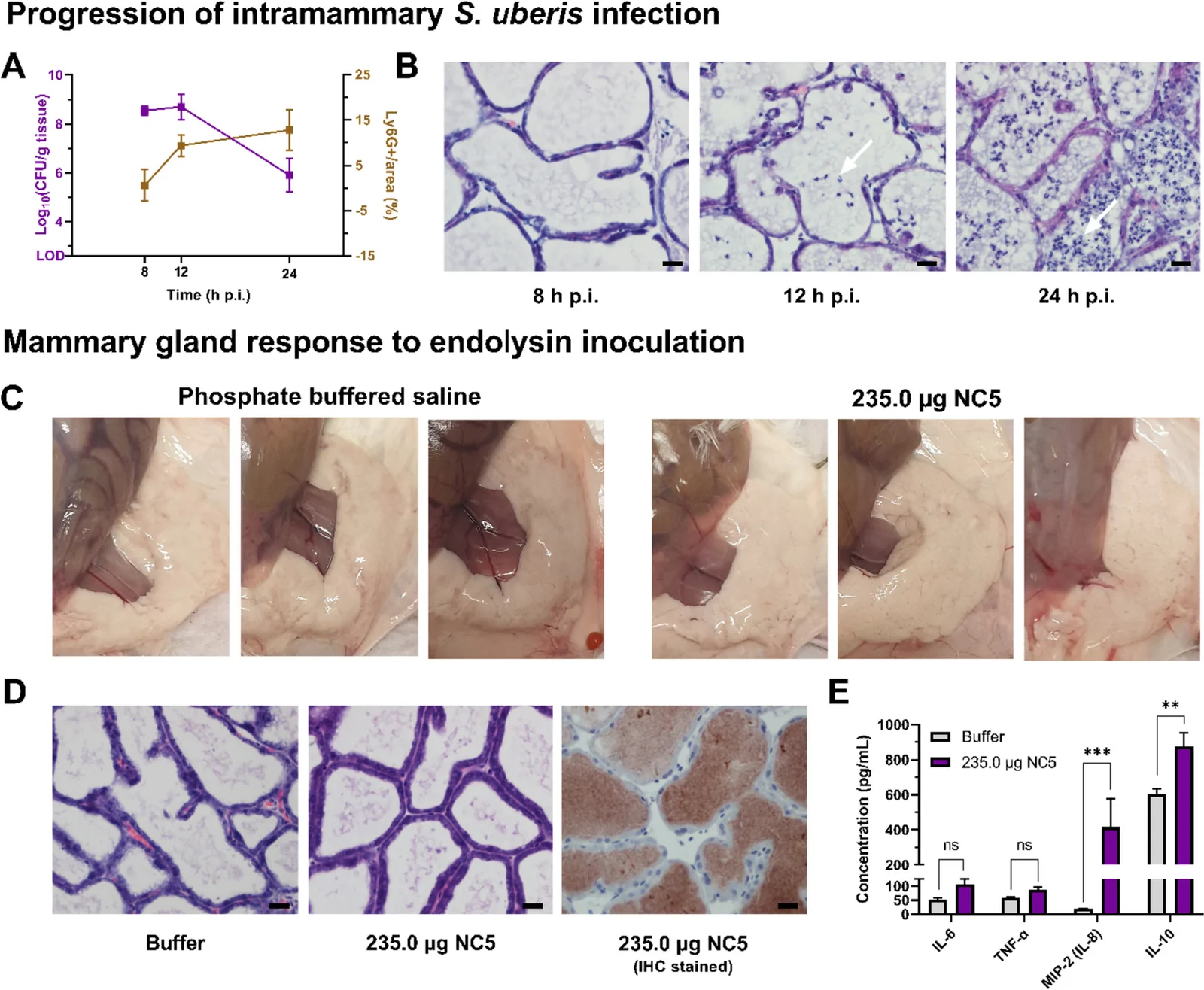
Progression of an intramammary *S. uberis* infection and determination of the bacterial load and neutrophil influx at 8, 12, and 24 h post-infection (p.i.), as well as the mammary gland response to an intraductal inoculation with either 235.0 μg endolysin NC5 or buffer (phosphate buffered saline). **A** Bacterial load (purple, left *y*-axis) and quantification of immunohistochemical Ly6G-positive neutrophil staining (brown, right *y*-axis). **B** Histopathological H&E–stained sections. White arrows indicate the presence of neutrophils in the mammary gland lumen. **C** Macroscopic evaluation of mammary glands treated with either endolysin buffer or 235.0 μg NC5. **D** H&E-stained sections of mammary glands treated with either buffer (phosphate buffered saline) or 235.0 μg NC5, as well as IHC staining for the polyhistidine-tag of the endolysin. **E** TNF-α, IL-6, MIP-2 (IL-8), and IL-10 quantification in lysates of mouse mammary glands harvested 1 h after an intraductal inoculation with either buffer or 235.0 μg NC5. LOD, limit of detection (400 CFU/g tissue). Data are shown as the mean and an error bar representing the standard error of the mean (SEM). Microscopic images were captured at a ×40 magnification, showcasing mammary glands of different mice. A scale bar, denoting 20 μm, provides a reference for measurement

Prior to evaluating the supplemental effect of endolysin NC5 to cloxacillin treatment, the progression of an intramammary *S. uberis* infection as well as the potential acute cytotoxicity of endolysin NC5 was evaluated in the mouse model for bovine mastitis (Fig. 1). Therefore, mice (*n* = 18) were inoculated intramammarily with 10^3^ CFU of a selected *S. uberis* field isolate belonging to the pathotype GGC ST-5, which our group previously isolated from a clinical bovine mastitis case (Vander Elst et al. 2023b). The bacterial load as well as the influx of neutrophils was subsequently evaluated at 8, 12, and 24 h post-infection (p.i.) (*n* = 6 per group). The isolate propagated in the murine mammary gland, as a bacterial load of (expressed as mean ± standard error of mean (SEM)) 8.54 ± 0.18, 8.70 ± 0.52, and 5.90 ± 0.68 log_10_(CFU/g tissue), was retrieved at these predetermined time points, respectively (Fig. 1A). An influx of neutrophils was observed at 12 h p.i. that further increased at 24 h p.i. (Fig. 1A, white arrows in Fig. 1B, and Fig. S1). Subsequently, the potential acute cytotoxicity of the selected high dose (235.0 μg) of endolysin NC5 was compared to mammary glands inoculated with endolysin buffer (phosphate buffered saline (PBS)) (*n* = 3 per group). Endolysin NC5 was purified from overexpression in *E. coli* BL21, and the endotoxin content thereof (0.25 EU/mL) was within acceptable limits for the preclinical evaluation of new biologicals (Malyala and Singh 2008). No abnormalities in behavior of the mice were observed at the point of euthanasia (i.e., after 1 h), and tissue irregularities were absent in all inoculated mammary glands upon necropsy (Fig. 1C). No difference between the endolysin- and buffer-treated mammary glands was observed as evaluated by histology, and proper intraductal administration of endolysin NC5 was confirmed by IHC for its polyhistidine-tag (Fig. 1D and Fig. S2). Local levels of IL-6 and TNF-α, which are general pro-inflammatory cytokines, were, respectively, 2.0- and 1.5-fold (*p* > 0.05 for both) increased in endolysin *vs.* buffer-treated mammary glands (Fig. 1E). Local levels of macrophage inflammatory protein-2 (MIP-2), the mouse IL-8 homologue and a hallmark of mastitis due to its neutrophil-chemotactic properties, were increased approximately 20-fold (*p* < 0.001) in endolysin *vs.* buffer-treated mammary glands (Fig. 1E). These MIP-2 data suggest together with the increased TNF-α and IL-6 levels that the selected high dose of 235.0 μg induces a preconditioning inflammatory response. However, a 1.5-fold (*p* < 0.01) increase of the anti-inflammatory cytokine IL-10 was also observed (Fig. 1E).

Following the initial characterization of *S. uberis* propagation in the mammary gland and considering the preconditioning inflammatory response of endolysin NC5, it was evaluated if cloxacillin treatment could be improved by supplementation of either a low or a high dose of endolysin NC5. This was specifically aimed at evaluating the mastitis hallmarks in the treated mammary glands. Therefore, mice were similarly intramammarily infected with the *S. uberis* field isolate and given treatment 12 h thereafter. This treatment consisted of 30.0 μg cloxacillin that was supplemented with endolysin NC5 in either a low (23.5 μg) or high (235.0 μg) dose. Additionally, as a means of control, mice were also treated with 30.0 μg cloxacillin as a stand-alone treatment or with buffer (PBS) as a placebo treatment. To thoroughly investigate significant effects between the cloxacillin stand-alone (*n* = 11) and high-dose combination treatment (*n* = 10), the sample sizes in these groups were intentionally increased in comparison to the low dose (*n* = 6) and placebo (*n* = 4) treatment, given that the latter group was already evaluated in the kinetic experiment at three different time points (Fig. 1A). Mice were euthanized at 16 h p.i. (i.e., 4 h post-treatment) to consciously detect small differences between the experimental groups mid-treatment.

**Fig. 2:**
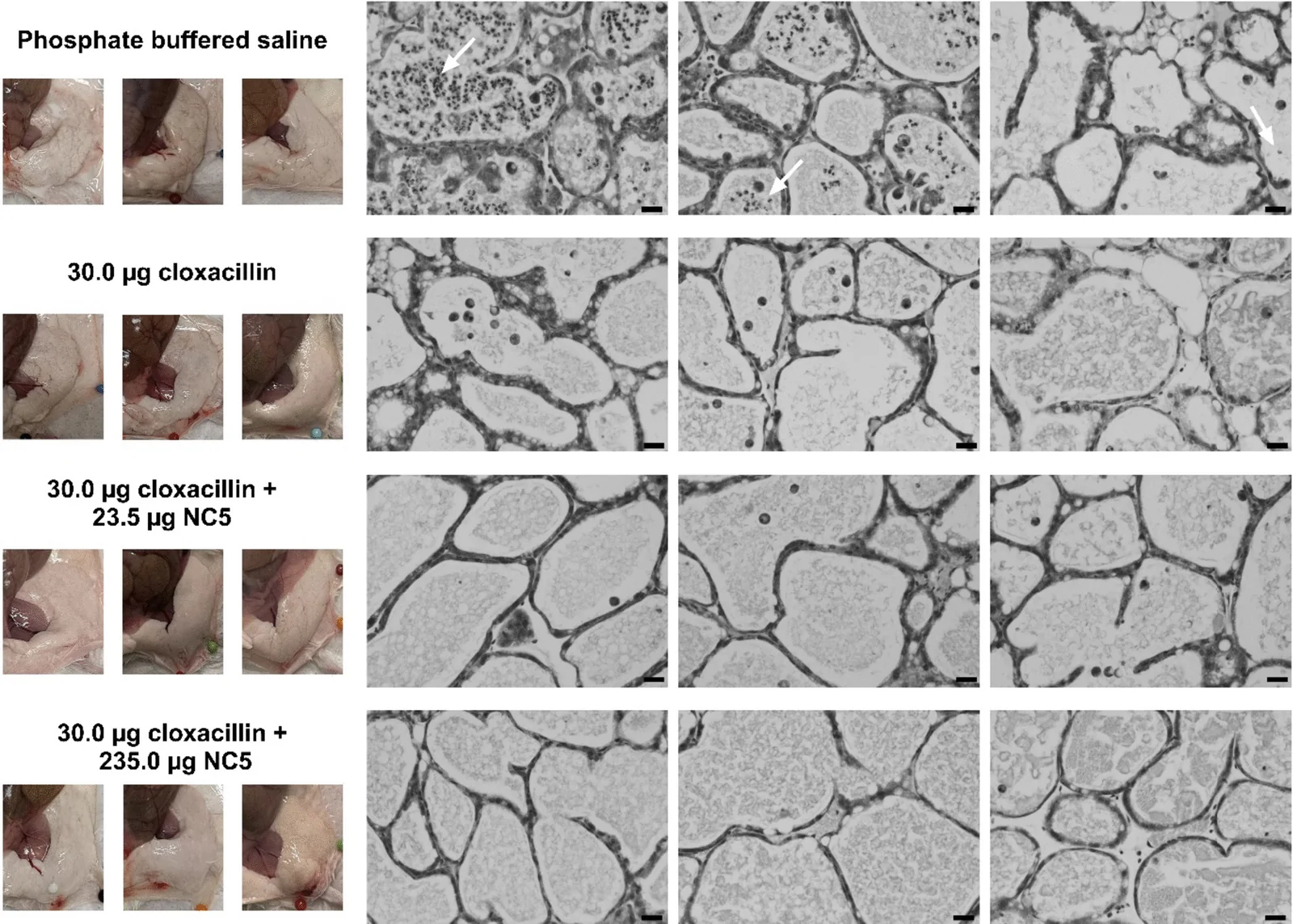
Macro- and microscopic evaluation of the hallmarks of mastitis in *S. uberis*–infected murine mammary glands following treatment. Cloxacillin treatment was supplemented with endolysin NC5 in either a low (23.5 μg) or a high (235.0 μg) dose and compared to cloxacillin monotherapy as well as placebo (phosphate buffered saline) treatment. White arrows indicate the presence of neutrophils in the murine mammary gland lumen. Microscopic images were captured at a ×40 magnification, showcasing mammary glands of different mice. The scale bar, denoting 20 μm, provides a reference for measurement

As a first hallmark, the foremost clinical symptoms of mastitis were evaluated in all mice. The mice did not develop fever and did not display abnormal behavior, nor pain at the injection site, during the 16-h course of the experiment. No macroscopic abnormalities in the mammary glands were observed upon necropsy (Fig. 2), except for one mouse of the high-dose combination therapy group that had swelling (i.e., edema), increased vascularization, and redness in both the left and right mammary gland (data not shown). Next, tissue sections were subjected to histopathological examination to evaluate the influx of neutrophils as a second hallmark of mastitis. This revealed a variable influx of neutrophils in the mammary glands of mice that received placebo treatment and was less pronounced in the group treated with cloxacillin monotherapy as well as the low-dose combination treatment (white arrows in Fig. 2). However, this influx clearly diminished upon the addition of a high dose of endolysin NC5 to cloxacillin. This observation was substantiated through the quantification of neutrophils via Ly6G-positive staining, which revealed a 1.8-, 2.0-, and 5.7-fold reduction (*p* > 0.05 for all) in the groups receiving antibiotic monotherapy, low-dose, and high-dose combination therapy compared to the placebo group, respectively (Fig. S3).

**Fig. 3:**
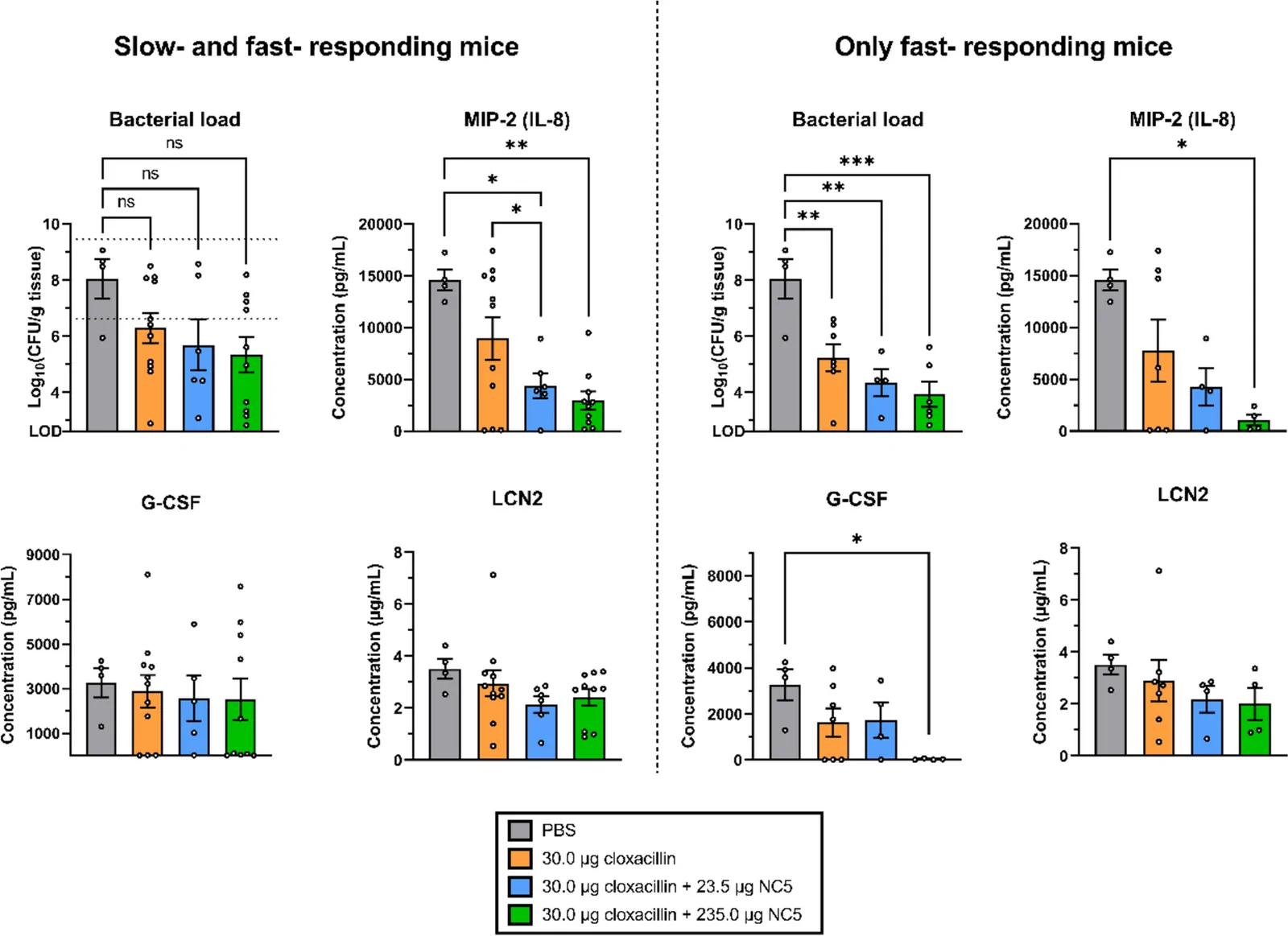
Comparative evaluation of the bacterial load and neutrophil-associated pro-inflammatory mediators MIP-2 (IL-8), G-CSF, and LCN2 between slow- and fast-responding mice and fast-responding mice alone. Data are shown as individual points representing each mouse with a bar indicating the mean and an error bar representing the standard error of the mean (SEM). The dotted line indicates the mean ± 2× SEM of the placebo group as a criterium to exclude presumed slow responding mice. Two mice in the group that received 30.0 μg cloxacillin supplemented with 235.0 μg NC5 consistently had outliers and were removed in the dataset concerning the fast-responding animals. Single (*) and double (**) asterisks indicate *p* < 0.05 and *p* < 0.01, respectively

The bacterial load, the third hallmark of mastitis, was reduced in the mammary glands at 16 h p.i. in both combination therapy groups compared to both control treatments (Fig. 3). The highest reduction in bacterial load was observed in the group receiving supplementation with the high endolysin dose. More specifically, stand-alone antibiotic treatment caused — on average — a 55-fold decrease (*p* > 0.05) of the bacterial load compared to the placebo group, whereas 225- to 500-fold decreases (*p* > 0.05 for both) were seen upon addition of the low and high endolysin dose, respectively. Unexpectedly, it was observed that the antibiotic stand-alone and both combination therapy groups showed a high variability indicative for two types of responders, i.e., mice that already showed a response after the 4-h treatment (*n* = 17) and mice that showed no clear response after 4-h treatment yet (*n* = 10) (Fig. 3). If these presumed slow-responding mice were excluded, based on the criterium that CFU levels were within the mean ± 2× SEM of the placebo group (i.e., 8.04 ± 1.42 log_10_(CFU/g tissue)), significance could be demonstrated for all treatment groups in comparison with the placebo control. More specifically, an average decrease of 630-, 4900-, and 13,000-fold (*p* < 0.01, *p* < 0.01, and *p* < 0.001) was observed in CFU numbers for the antibiotic stand-alone, the low-dose, and high-dose combination therapy groups in these presumed fast responders, respectively.

As a fourth hallmark of mastitis, the local inflammatory protein profile was measured on mammary gland lysates with particular attention to the neutrophil-associated markers MIP-2 (IL-8), G-CSF, and LCN2 (Fig. 3). Supporting the decrease in neutrophil influx as immunohistochemically observed, the local concentration of the neutrophil chemokine MIP-2 showed a dose-dependent reduction when NC5 was added to cloxacillin. More specifically, a significant 3.3- and 5.0-fold decrease for MIP-2 (*p* < 0.01 for both) was observed comparing the low- and high-dose combination therapy groups with the placebo group, respectively. Only a 1.6-fold decrease for MIP-2 (*p* > 0.05) was observed for the cloxacillin treatment group compared to the placebo group. An 8.5-fold (*p* < 0.05) difference in MIP-2 levels was observed between the antibiotic monotherapy and high-dose combination therapy groups. In line with these findings for MIP-2, local levels of the neutrophil-maturating and neutrophil-activating cytokine G-CSF and the neutrophil-associated antimicrobial iron-chelating protein LCN2 showed a similar downward trending, albeit better distinguished for the presumed fast-responding mice (Fig. 3, right panel). More specifically, the antibiotic stand-alone, low-dose, and high-dose combination therapy groups showed a 2.0-, 1.9-, and 93.7-fold G-CSF decrease (*p* > 0.05, *p* > 0.05, and *p* < 0.05) in comparison with the placebo group, respectively. For LCN2, this reduction was 1.2-, 1.3-, and 1.6-fold (*p* > 0.05 for all). Surprisingly, addition of a high dose of NC5 did not further decrease the G-CSF and LCN2 levels if the presumed slow responding mice were also considered. Moreover, local concentrations of LCN2 trended upward slightly in the high- *vs.* low-dose combination therapy groups. Collectively, these findings imply that the slow- in contrast to the fast-responding mice still exhibit an elevation in neutrophil-associated markers as a response to the increased levels of propagating *S. uberis* at 16 h p.i. (Fig. 3, left panel).

**Fig. 4:**
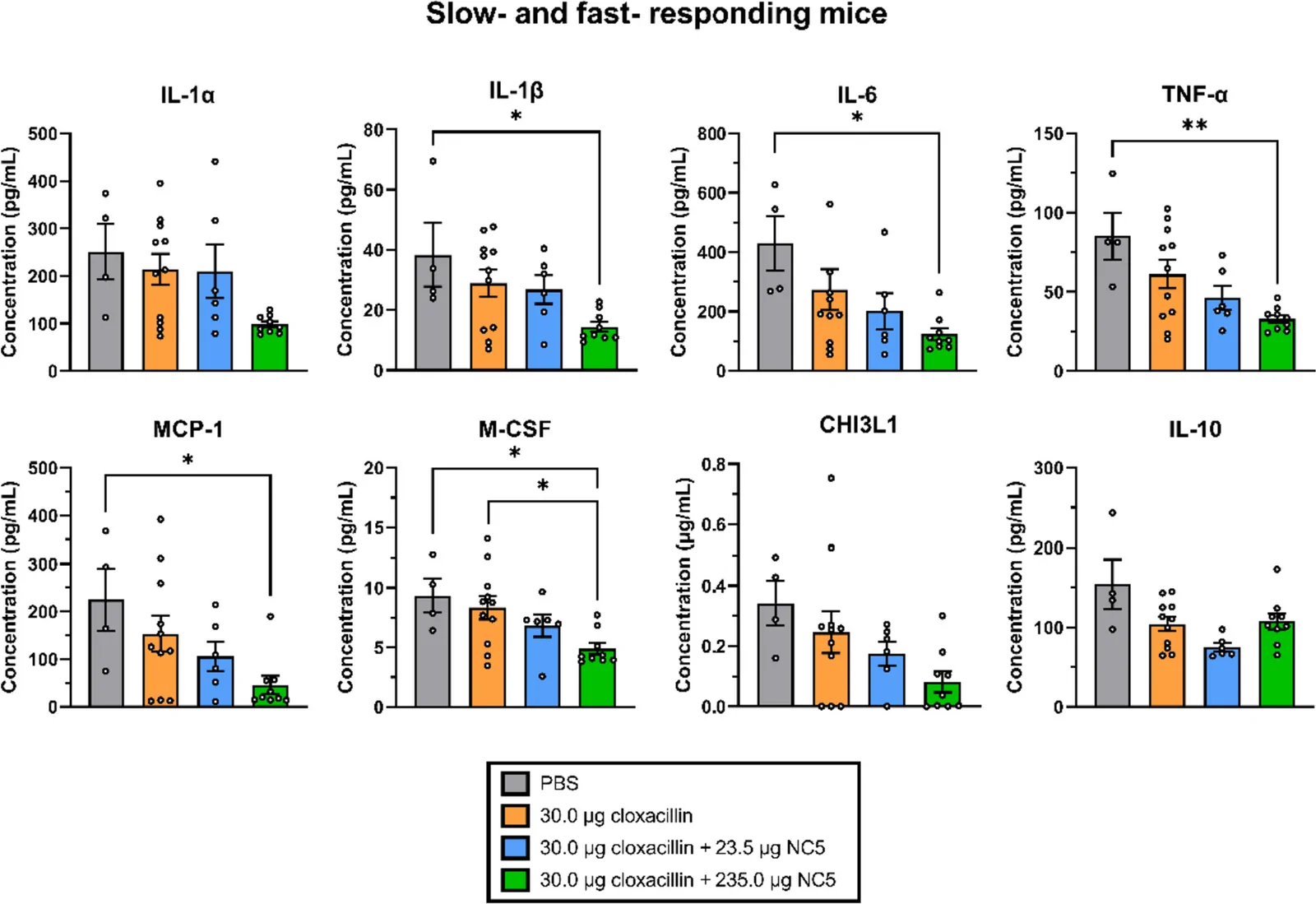
Comparative evaluation of the local inflammatory protein profile between the different treatment groups of this study, including both slow- and fast-responding mice. Data are shown as individual points representing each mouse with a bar indicating the mean and an error bar representing the standard error of the mean (SEM). One mouse in the group that received 30.0 μg cloxacillin supplemented with 235.0 μg NC5 consistently had outliers and was removed from the dataset. Single (*) and double (**) asterisks indicate *p* < 0.05 and *p* < 0.01, respectively

The local immune profile was further complemented by the evaluation of other pro-inflammatory cytokines, chemokines, growth factors, and metabolites (Fig. 4). This revealed an overall dose-dependent reduction caused by supplementation of endolysin NC5 to cloxacillin. Interestingly, this effect was observed regardless of the exclusion of the slow-responding mice, indicating that mice with a lack of reduction in bacterial load still exhibited an immunological response at 16 h p.i. for the markers that were hereafter evaluated (Fig. 4 and Fig. S4). Starting with the general pro-inflammatory mediators IL-1α, IL-1β, IL-6, and TNF-α, the local levels decreased 2.4-, 2.4-, 4.1-, and 2.4-fold (*p* > 0.05, *p* < 0.05, *p* < 0.05, and *p* < 0.01) in the high-dose combination therapy group in comparison with the placebo group, whereas these decreases were 1.2-, 1.4-, 2.2-, and 1.8-fold (*p* > 0.05 for all) for the low-dose combination therapy group, and 1.2-, 1.3-, 2.0-, and 1.4-fold (*p* > 0.05 for all) for the cloxacillin stand-alone group, respectively. The local levels of the macrophage-associated inflammatory mediators MCP-1 and M-CSF showed similar dose-dependent reductions after addition of endolysin NC5 to cloxacillin. More specifically, levels of MCP-1 and M-CSF trended downward slightly 1.5-fold and 2.1-fold (*p* > 0.05 for both) and were significantly reduced 8.0-fold (*p* < 0.05) for MCP-1, as well as 1.1-, 1.4-, and 1.9-fold (*p* > 0.05, *p* > 0.05, and *p* < 0.05) for M-CSF, respectively, for the antibiotic stand-alone, low-dose, and high-dose combination therapy groups in comparison with the placebo group. Like for the neutrophil attractant MIP-2, there was a 1.8-fold difference in M-CSF levels between the antibiotic stand-alone and the high-dose combination therapy groups (*p* < 0.05). In addition, to evaluate the influx of circulating macrophages in the treated murine mammary glands, IHC for Iba-1 was performed. This staining showed the moderate presence of mammary ductal macrophages that physiologically reside between the luminal and basal epithelial cells of the lactating mammary gland, but no differences in the number of macrophages between the treatment groups were observed (*p* > 0.05; Fig. S5). Finally, local levels of the innate immunity marker CHI3L1 were determined. Supplementation of NC5 to cloxacillin again resulted in a dose-dependent trending downward of the levels of CHI3L1 by 1.4-, 2.0-, and 3.6-fold (*p* > 0.05 for all) for the antibiotic stand-alone, low-dose, and high-dose combination therapy groups in comparison with the placebo group, respectively. Of note, the local levels of the anti-inflammatory cytokine IL-10 were 1.5- and 2.1-fold reduced (*p* > 0.05 and *p* < 0.01) for cloxacillin as stand-alone and the low-dose combination therapy in comparison with the placebo group, respectively. Corroborating the observation for IL-10 upon evaluating 235.0 μg NC5 regarding the exclusion of its potential acute cytotoxicity at 1 h post-treatment, local concentrations of IL-10 still trended upward slightly by 1.4-fold in the high- *vs.* low-dose combination therapy group, but were decreased 1.5-fold compared to the placebo group (*p* > 0.05 for both).

## Discussion

Lactating mice are a valid preclinical tool to screen novel antimicrobial compounds for mastitis, such as endolysins (Schmelcher et al. 2012, 2015; Becker et al. 2016; Gutiérrez et al. 2020; Liu et al. 2022). The use of mice as an elegant model for dairy cows comes with certain limitations and strengths, as reviewed in Notebaert and Meyer (2006) and Ingman et al. (2015). Relying on a mouse model for *S. uberis* mastitis, the aim of this study was to investigate if the previously *in vitro* characterized antimicrobial potential of the VersaTile engineered endolysin NC5 could be extended to an *in vivo* setting, specifically as a supplemental compound to intramammary cloxacillin treatment. The unexpected observation of slow- and fast-responding animals was not considered in the initial study design, resulting in sample sizes that were suboptimal to demonstrate overall significant treatment differences between these responder subgroups. Nevertheless, the study aim was partly attained given that the supplementation of endolysin NC5 in comparison to cloxacillin as a monotherapy dose-dependently reduced the bacterial load faster (albeit only significantly in the fast-responding animals), mitigated the influx of neutrophils, and was marked by a decrease in local pro-inflammatory mediators.

The division into slow- and fast-responding animals resembles an observation made in a similar study in which the outcomes of an intramammary infection following experimental *E. coli* challenge were studied in dairy cows (Messom et al. 1993). The authors categorized individual animals into moderate or severe responders according to cow-specific factors (Burvenich et al. 2003). These moderate responders were characterized by a rapid (< 24 h) milk somatic cell count (SCC) increase that limited the intramammary propagation of *E. coli* as well as the mastitis symptoms. Conversely, the severe responders were identified by a delayed (> 24 h) SCC elevation that resulted in substantial *E. coli* proliferation as well as mastitis symptoms. We infer that these moderate responders may correspond to the presumed fast responder mice of our study and the severe responders to the presumed slow responder mice. Although direct measurement of SCC in mouse milk is not feasible, influx of neutrophils can indirectly be evaluated by an increase of the neutrophil-associated markers MIP-2, G-CSF, and LCN2. These latter markers, and in particular G-CSF, remained elevated in the total population because of the presumed slow (severe) responders during the high-dose combination therapy. Thus, these presumed slow (severe) responders are marked by an ongoing increased neutrophil-targeted signaling to still tackle the intramammary bacterial load that remains elevated. In sharp contrast, a reduction of MIP-2, G-CSF, and LCN2 levels as well as of neutrophil influx (assessed through histology and confirmed by immunohistochemical Ly6G quantification) was observed in all the presumed fast (moderate)-responding mice for the high-dose combination treatment. Taking together, these observations suggest that two different biological subgroups were indeed present in the current study.

An explanation for this unexpected division into slow- *vs.* fast-responding animals might lie in the rational choice for a short (4 h) duration of the treatment and the deliberate use of outbred mice (Woodward and Whittem 2019). Cloxacillin, being an isoxazolyl penicillin, needs to be incorporated into the cell wall of multiplying Gram-positive bacteria to effectively eliminate them (Kim et al. 2023). As a result, the CFU levels in the cloxacillin treatment groups, with or without addition of endolysin NC5, were as expected not completely reduced to the limit of detection after only 4 h. Nevertheless, in our previous *in vitro* study, an accelerated reduction in *S. uberis* CFUs was observed in raw milk between the 4 and 8 h time points for the supplemental strategy (Vander Elst et al. 2023b). Therefore, in this preclinical *in vivo* study, we deliberately choose to evaluate the combination treatment after only 4 h to specifically capture the small discrepancies between the experimental groups mid-treatment (Woodward and Whittem 2019). However, this limits the current study to one early endpoint and does not provide a comprehensive kinetic profile to allow a more holistic interpretation of the obtained results. The second deliberate choice of outbred instead of inbred mice most likely further contributes to the observed occurrence of two types of responding animals. Indeed, inbred mice typically exhibit a more consistent and uniform response due to their genetic homogeneity (Aldinger et al. 2009). However, they may not accurately represent the genetic diversity present in dairy herds. By using outbred mice in this study and in the previous preclinical studies from our research group, we aim to capture a broader range of genetic variations that better reflect field conditions (Festing 2010).

Similar to the outcome of this study, the preclinical efficacy of endolysins in mouse models for bovine mastitis has previously been reported for both wild-type and engineered endolysins other than NC5 (Schmelcher et al. 2012, 2015; Becker et al. 2016; Gutiérrez et al. 2020; Liu et al. 2022). Synergy between another isoxazolyl penicillin (i.e., oxacillin) and another engineered endolysin (i.e., ClyS) was previously reported once in the context of murine nasal decolonization for methicillin-resistant *Staphylococcus aureus* (Daniel et al. 2010). A key difference between the current study and those from other groups investigating the antimicrobial effects of endolysins in preclinical models for bovine mastitis is the timing of treatment relative to the establishment of infection. Other groups administered endolysins at a very early time point, i.e., already within 1 h after intramammary inoculation of the bovine udder pathogen (Schmelcher et al. 2012, 2015; Liu et al. 2022). Only one study reported the administration of an endolysin 6 h after inoculating *S. aureus* (Becker et al. 2016). The validity of preclinical models employing such short time intervals between pathogen inoculation and treatment can be questioned, as the activation of the mammary gland innate immunity is not yet fully initiated at that time of treatment. In the current study, an influx of neutrophils was consistently observed at 12 h after intramammary inoculating the selected *S. uberis* isolate, which is in line with previous findings for *S. uberis* as well as other mastitis pathogens (Breyne et al. 2017, 2018; Salamon et al. 2023; Vander Elst et al. 2020a, 2023a). More importantly and from a translational point of view, dairy farmers typically consider therapeutic treatment of their animals only when an increase in the cow’s milk SCC is detected, which does not occur within 1 h after *S. uberis* invades the mammary gland (Johnzon et al. 2018; Rainard et al. 2022; Lipkens et al. 2023). Therefore, these previous studies may have missed certain interactions and impacts originating from the mammary gland innate immunity on the *in vivo* functionality of their evaluated endolysins (Murray et al. 2021). This is particularly important for the investigated major pathogen, as *S. uberis* is known to evade recognition by the mammary gland epithelium, resulting in a delayed and reduced inflammatory response compared to other mastitis-causing pathogens (Günther et al. 2016; Rainard et al. 2022). In line with these reports, it is worth noting that all *S. uberis*–infected mice — except for one — showed the absence of clinical symptoms and macroscopic abnormalities in the infected mammary glands for the selected isolate. This early innate immune evasion by *S. uberis* may be mitigated through the intramammary administration of endolysin NC5. Indeed, our data suggest a preconditioning effect on the healthy mammary gland, marked by a significant increase in the neutrophil chemoattractant MIP-2 (i.e., IL-8), albeit from a far lower order of magnitude than the overt innate immune response caused by *S. uberis* infection. Our research group has previously demonstrated for other compounds that such a preconditioning effect can protect the mammary gland against the subsequent proliferation of major mastitis pathogens, including *S. uberis* (Breyne et al. 2017; Vander Elst et al. 2023a). Notably, it was also observed that the levels of the anti-inflammatory protein IL-10 increased upon inoculation with a high dose of endolysin NC5 compared to a low dose, but this should be interpreted with caution as IL-10 also plays a crucial role in supporting proper milk production and lactation (Ezzat Alnakip et al. 2014).

In conclusion, this study identified two distinct types of responder mice following intramammary cloxacillin treatment, irrespective of endolysin NC5 supplementation. Fast-responding mice demonstrated enhanced early cloxacillin treatment when supplemented with endolysin NC5, leading to diminished *S. uberis* propagation, reduced neutrophil influx, and a dose-dependent decrease in local pro-inflammatory mediators. In contrast, the overall population exhibited unaltered levels of neutrophil-associated markers, attributed to presumed slow-responding mice, that were accompanied by an increased presence of intramammary *S. uberis* comparable to placebo treatment. This preclinical *in vivo* proof-of-concept, coupled with previous findings in mastitic milk from *S. uberis*–infected cows, establishes a robust foundation for further exploration of NC5 as a potential adjunct to cloxacillin for the — repeated — intramammary treatment of *S. uberis*–infected bovine udders.

## Supplementary information

ESM 1

## Data Availability

All data generated or analyzed during this study are included in this published article or can be provided upon reasonable request.
