# Supplementary material for: Endolysin NC5 improves early cloxacillin treatment in a mouse model of Streptococcus uberis mastitis
Source: Appl Microbiol Biotechnol. 2024 Jan 10;108:118. doi: 10.1007/s00253-023-12820-w (PMC10781846; doi:10.1007/s00253-023-12820-w)

Supplementary materials

Journal Name: Applied Microbiology and Biotechnology

Manuscript Title: Endolysin NC5 improves early cloxacillin treatment in a mouse model of *Streptococcus uberis* mastitis

Authors: Niels Vander Elst[1,2,3,*], Julie Bellemans[2,3], Rob Lavigne[1], Yves Briers[2] and Evelyne Meyer[3,#]

[1]Laboratory of Gene Technology, Department of Biosystems, Faculty of Bioscience Engineering, KU Leuven, Kasteelpark Arenberg 21, 3001 Heverlee, Belgium.

[2]Laboratory of Applied Biotechnology, Department of Biotechnology, Faculty of Bioscience Engineering, Ghent University, Valentin Vaerwyckweg 1, 9000 Gent, Belgium.

[3]Laboratory of Biochemistry, Department of Veterinary and Biosciences, Faculty of Veterinary Medicine, Ghent University, Salisburylaan 133, 9820 Merelbeke, Belgium.

*E-mail address of the corresponding author: Niels.VanderElst@ugent.be and niels.vander.elst@ki.se; telephone number of the corresponding author: +32 494 90 10 64.

Figure S1. Histopathological sections of murine mammary glands stained for Ly6G (neutrophils) in response to the intraductal inoculation with approximately 10^3 CFU of a clinical bovine mastitis-derived *S. uberis* isolate. Mammary glands were harvested 8, 12 and 24 h post-infection (p.i.). Pictures were captured at a 40× magnification, showcasing mammary glands of different mice. A scale bar, denoting 20 µm, provides a reference for measurement.

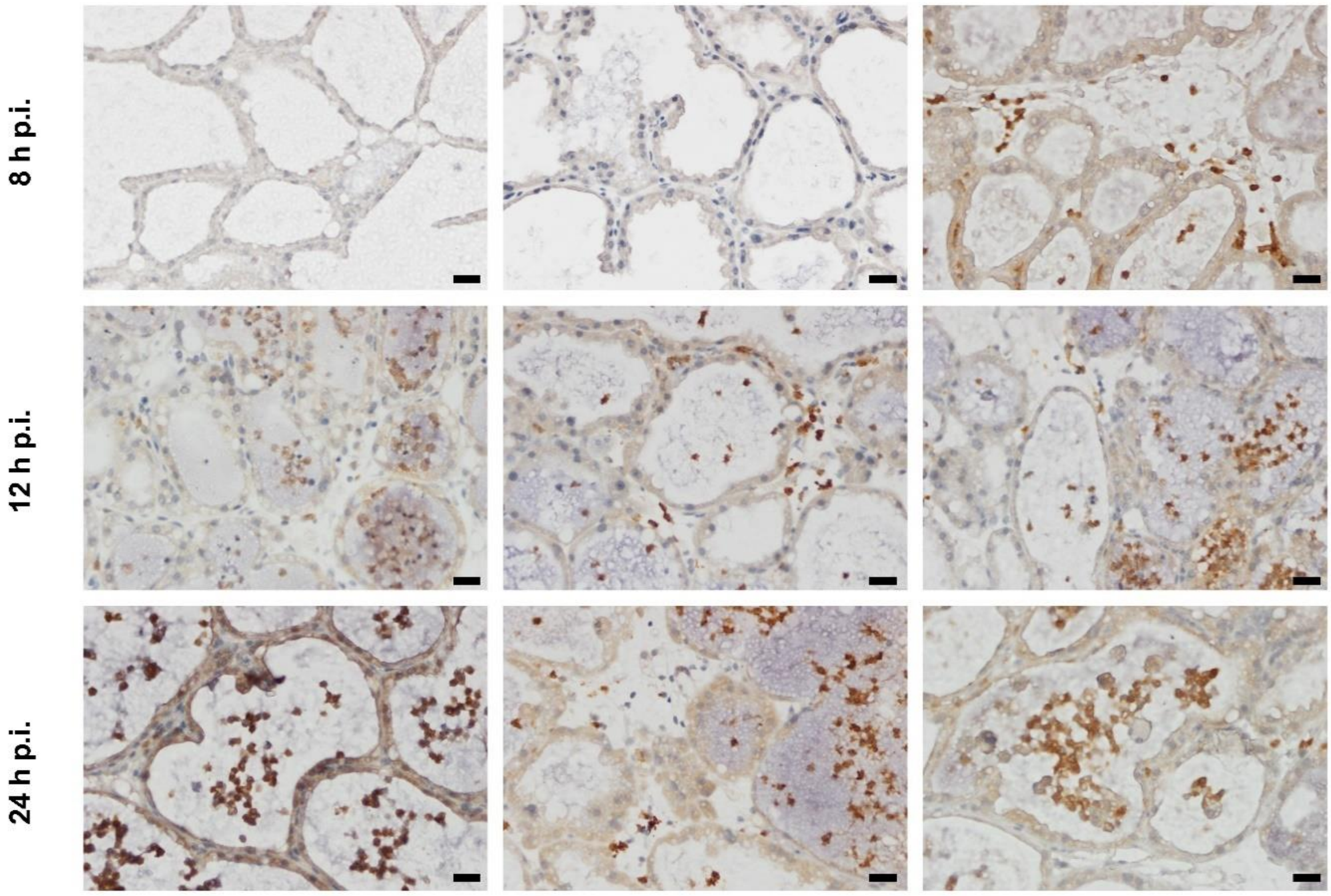

Figure S2. Histopathological sections of murine mammary glands stained for the polyhistidine tag of the endolysin after an intraductal inoculation with either 235.0 µg NC5 or endolysin buffer (phosphate buffered saline, PBS). Mammary glands were harvested 1 h post-inoculation. Pictures were captured at a 40× magnification, showcasing mammary glands of different mice. A scale bar, denoting 20 µm, provides a reference for measurement.

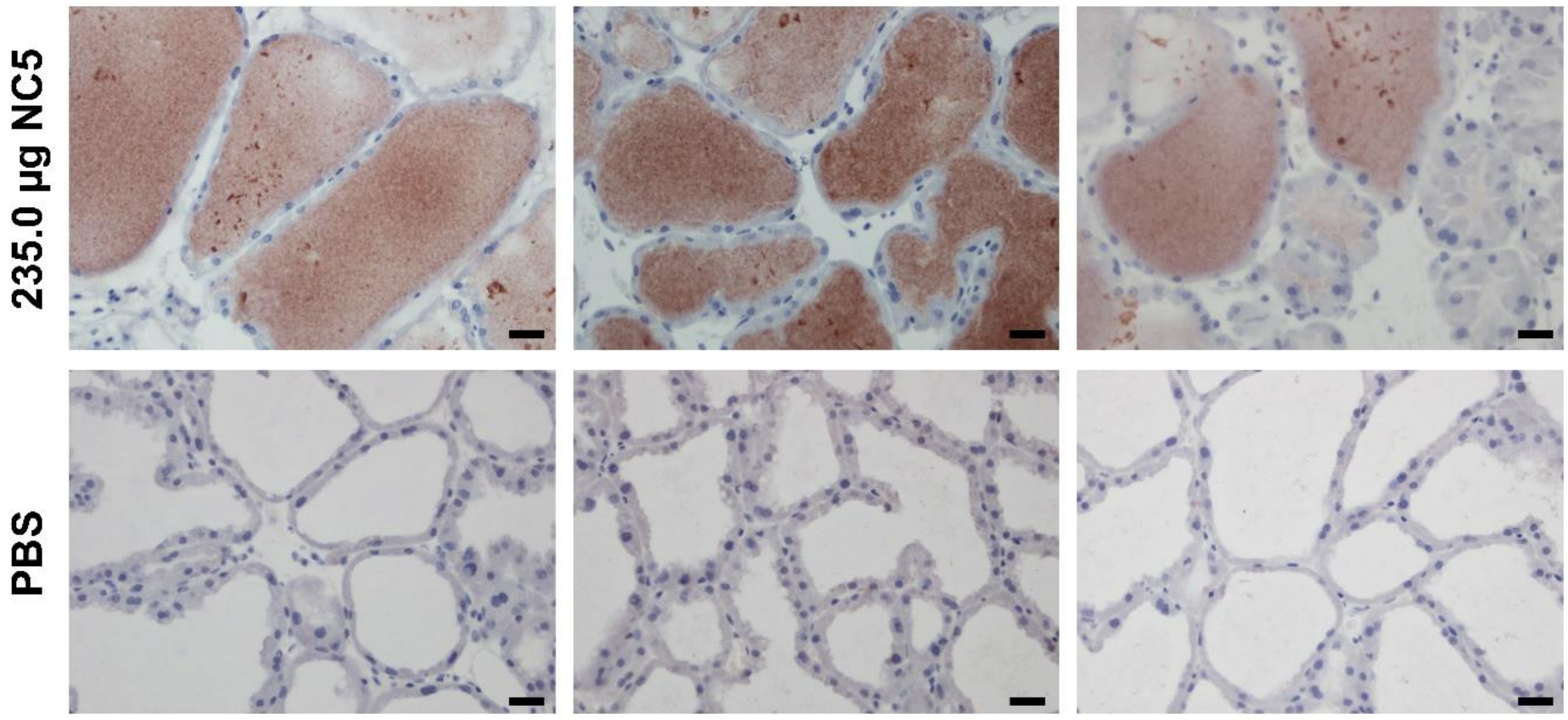

Figure S3. Histopathological sections and quantification of murine mammary glands stained for Ly6G (neutrophils) in response to the intraductal inoculation with approximately 10^3 CFU *S. uberis* and treatment administered 12 h post-infection, consisting of either placebo (PBS), 30.0 µg cloxacillin as stand-alone antibiotic therapy, and addition of NC5 in a low (23.5 µg) and high (235.0 µg) dose to 30.0 µg cloxacillin as a combination therapy. Pictures were captured at a 40× magnification, showcasing mammary glands of different mice. A scale bar, denoting 20 µm, provides a reference for measurement.

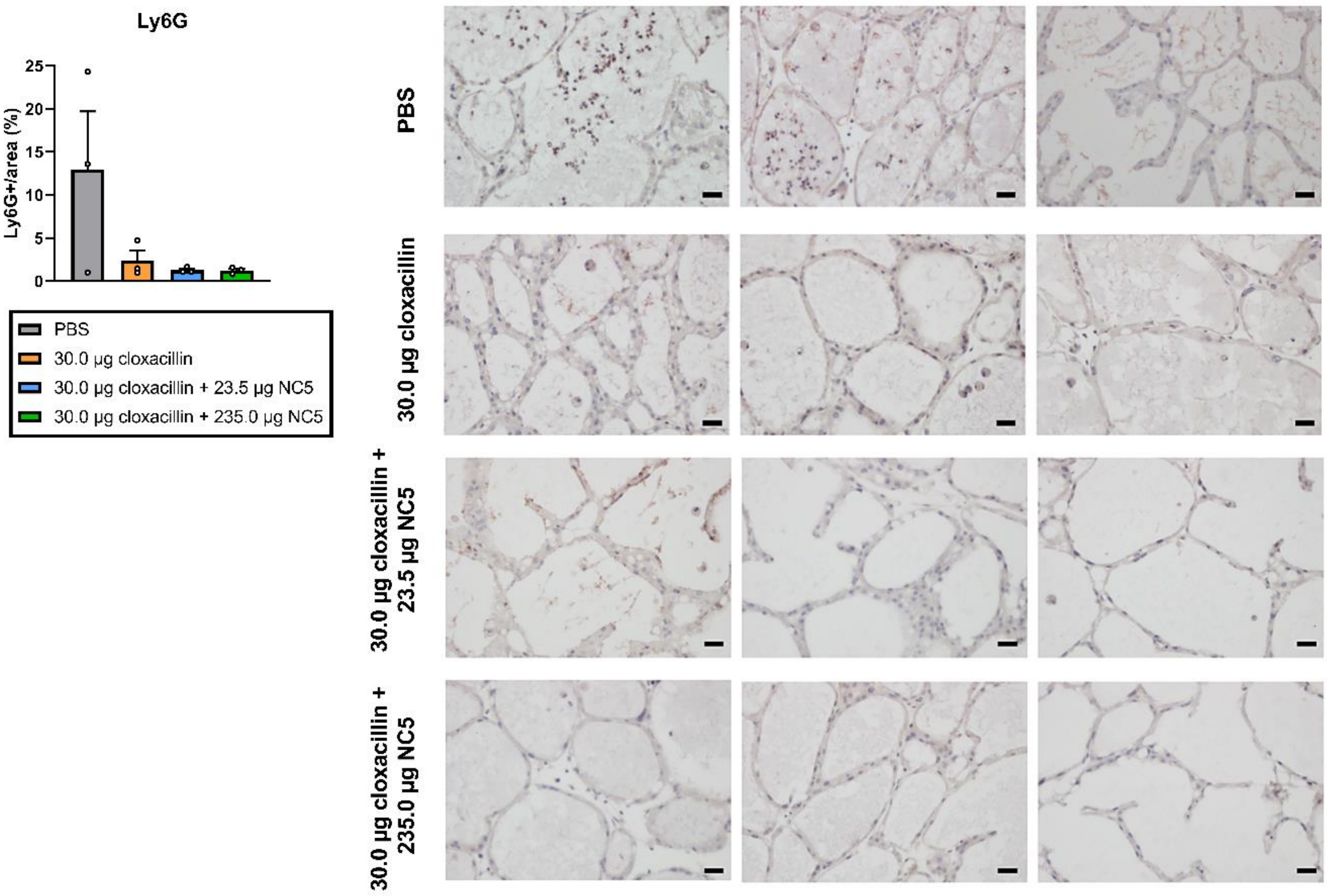

Figure S4. Comparative evaluation of the inflammatory protein profile between the different treatment groups of this study after exclusion of the slow-responding mice. Data are shown as individual points representing each mouse with a bar indicating the mean and an error bar representing the standard error of the mean. One mouse in the group that received 30.0 µg cloxacillin supplemented with 235.0 µg NC5 consistently had outliers and was removed from the dataset. A single (*) asterisk indicates $p < 0.05$.

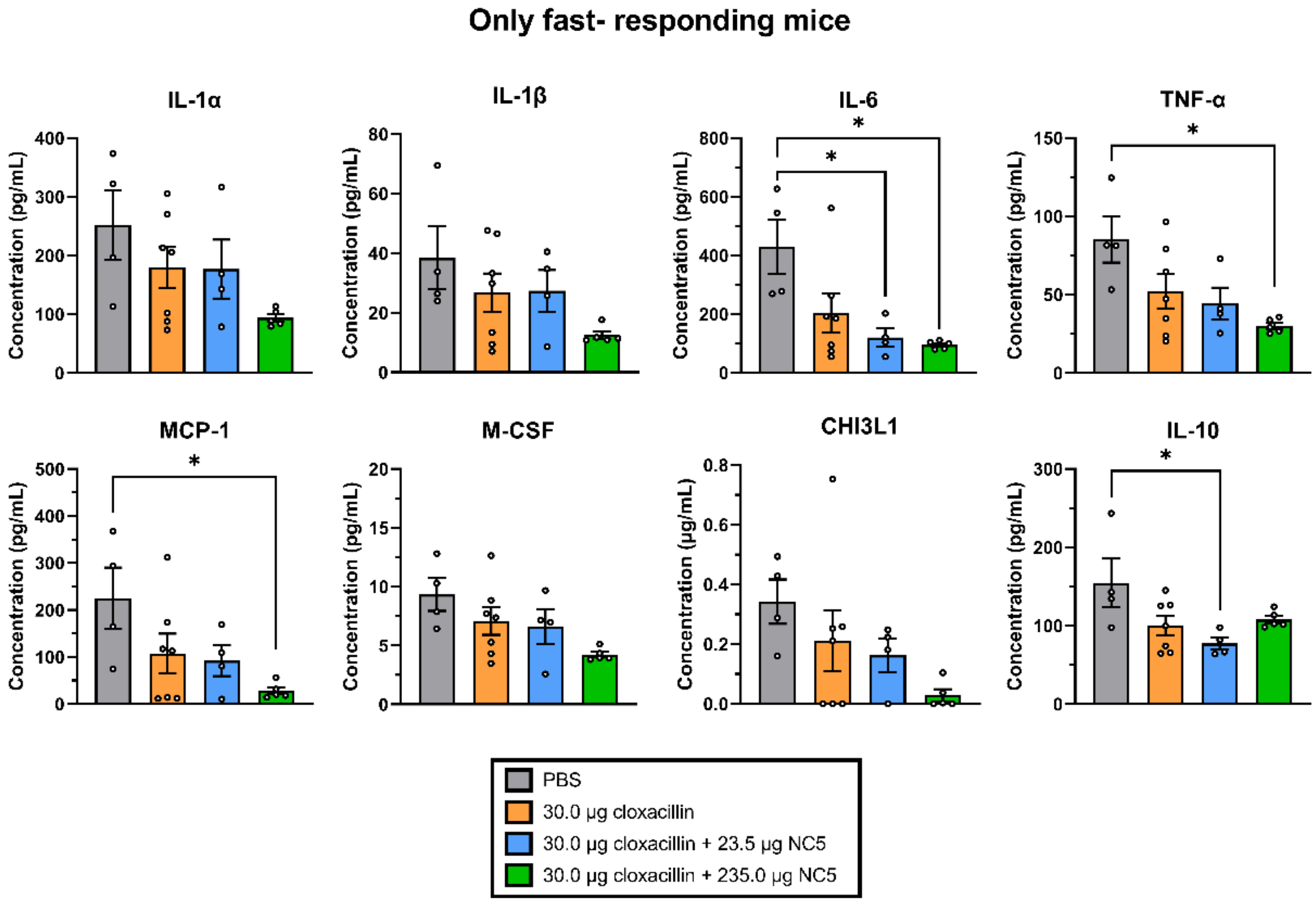

Figure S5. Histopathological sections and quantification of murine mammary glands stained for Iba-1 (macrophages) in response to an intraductal inoculation with 10^3 CFU *S. uberis* and treatment administered 12 h post-infection, consisting of either placebo (PBS), 30.0 µg cloxacillin as stand-alone antibiotic therapy, and NC5 in a low (23.5 µg) and high (235.0 µg) dose to 30.0 µg cloxacillin as a combination therapy. Pictures were captured at a 40× magnification, showcasing mammary glands of different mice. A scale bar, denoting 20 µm, provides a reference for measurement.

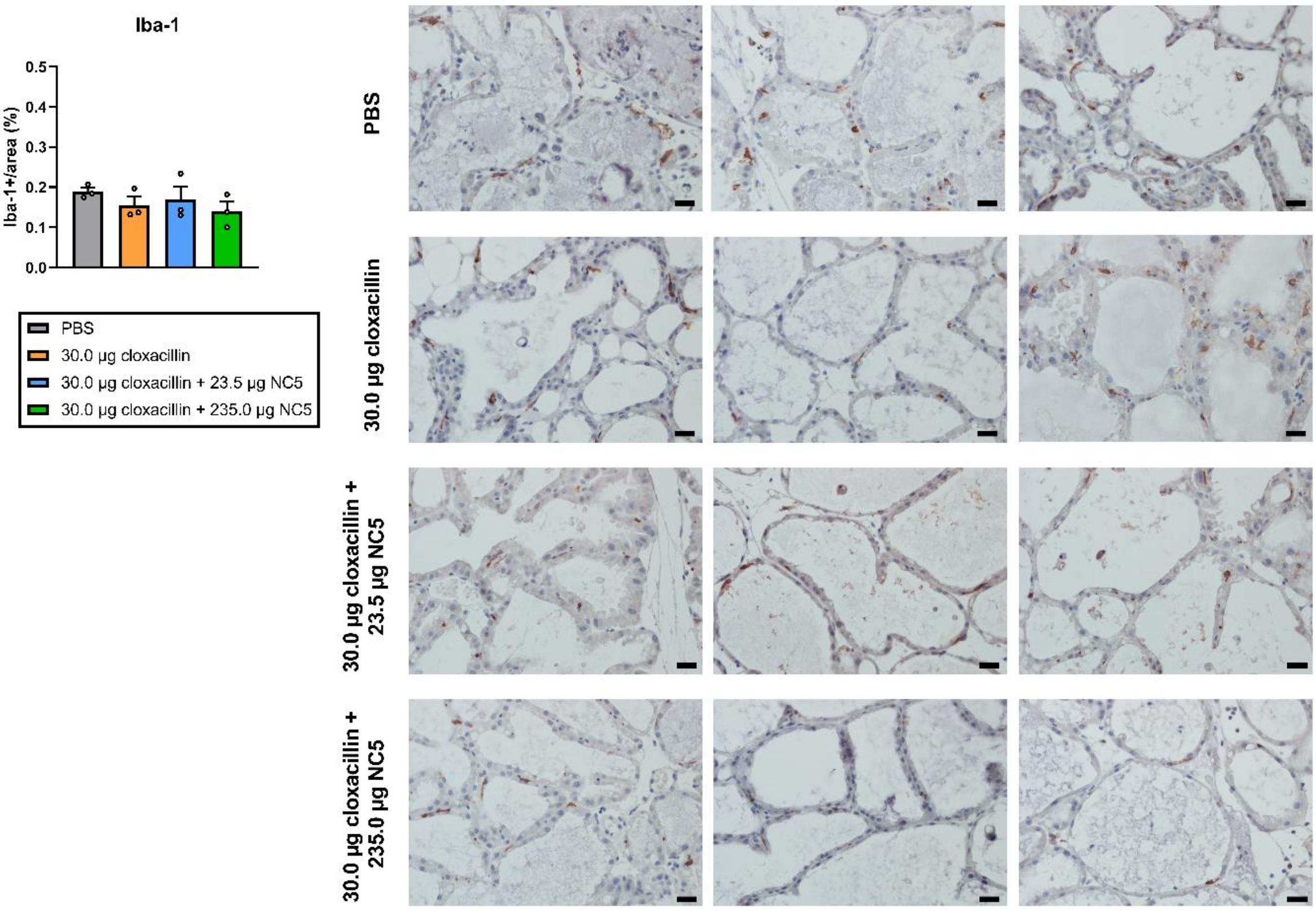

Supplement: Supplementary file 1 [file 253_2023_12820_MOESM1_ESM.pdf]
